# Soft Origami Optical-Sensing Actuator for Underwater Manipulation

**DOI:** 10.3389/frobt.2020.616128

**Published:** 2021-03-10

**Authors:** Zhong Shen, Yafei Zhao, Hua Zhong, Kailuan Tang, Yishan Chen, Yin Xiao, Juan Yi, Sicong Liu, Zheng Wang

**Affiliations:** ^1^ Department of Mechanical Engineering, The University of Hong Kong, Hong Kong, China; ^2^ Department of Computer Science, The University of Hong Kong, Hong Kong, China; ^3^ Department of Mechanical and Energy Engineering, Southern University of Science and Technology, Shen Zhen, China

**Keywords:** soft robotics, origami, actuator, optical sensing, underwater manipulation

## Abstract

Soft robots are ideal for underwater manipulation in sampling and other servicing applications. Their unique features of compliance, adaptability, and being naturally waterproof enable robotic designs to be compact and lightweight, while achieving uncompromized dexterity and flexibility. However, the inherent flexibility and high nonlinearity of soft materials also results in combined complex motions, which creates both soft actuator and sensor challenges for force output, modeling, and sensory feedback, especially under highly dynamic underwater environments. To tackle these limitations, a novel Soft Origami Optical-Sensing Actuator (SOSA) with actuation and sensing integration is proposed in this paper. Inspired by origami art, the proposed sensorized actuator enables a large force output, contraction/elongation/passive bending actuation by fluid, and hybrid motion sensing with optical waveguides. The SOSA design brings two major novelties over current designs. First, it involves a new actuation-sensing mode which enables a superior large payload output and a robust and accurate sensing performance by introducing the origami design, significantly facilitating the integration of sensing and actuating technology for wider applications. Secondly, it simplifies the fabrication process for harsh environment application by investigating the boundary features between optical waveguides and ambient water, meaning the external cladding layer of traditional sensors is unnecessary. With these merits, the proposed actuator could be applied to harsh environments for complex interaction/operation tasks. To showcase the performance of the proposed SOSA actuator, a hybrid underwater 3-DOFs manipulator has been developed. The entire workflow on concept design, fabrication, modeling, experimental validation, and application are presented in detail as reference for wider effective robot-environment applications.

## Introduction

Fast-growing interest in effective robot-environment interactions stimulated global investigations on soft robotics. The inherent compliance, light weight, and low cost of soft robots facilitated its development for various applications (Rus and Tolley, 2015; Wang et al., 2015). In particular, soft actuators, with their superior inherent adaptability, flexibility, and waterproof nature, are ideal for underwater manipulation to interact with various marine creatures and work in harsh environments (Calisti et al., 2011; Cianchetti et al., 2011; Arienti et al., 2013; Stilli et al., 2014; Stuart et al., 2014; Cianchetti et al., 2015; Giannaccini et al., 2018). Compared to the conventional rigid-bodied robots (Hamner et al., 1975; Xu et al., 2014), soft robotsoffer a totally new actuation and control approach to substitute the relatively bulky hardware and complicated control strategies. Constructive efforts on investigating the continuum and biomimetic motions with continuum mechanisms (Calisti et al., 2011; Cianchetti et al., 2011; Arienti et al., 2013; Stuart et al., 2014; Cianchetti et al., 2015) and Fluidic elastomer actuators (FEAs) (Stilli et al., 2014; Giannaccini et al., 2018) have proven the superior performance of soft actuators in underwater or in air applications.

However, the growing demand for use in wider and harsher applications has also stimulated soft robots to be more intelligent and robust, especially in harsh environments where higher manipulative capabilities and integrative robotic systems are vital. To further facilitate the high performance of soft robots, including a more integrative actuating-sensing module, larger output force, and more reliable sensory feedback, the scope of this study mainly focuses on exploring the actuation and sensing on a soft actuator level and soft sensor level.

Firstly, at the actuator level, popular soft actuators are made with flexible materials or flexible structures, such as shape memory alloys (SMAs) (Kode and Cavusoglu, 2007), electroactive polymers (EAPs) (Carpi et al., 2007), and fluidic elastic polymers (FEAs) (Stilli et al., 2014; Giannaccini et al., 2018). In particular, fluidic soft actuators have a fast response and multiple motion patterns, which are fabricated by a molding process with soft elastic polymers. However, the soft elastic materials limit the further progress of robots to a larger force output with plenty of energy wasted in materials’ inflation. In addition, the materials’ inflation brings non-linearity and hard-to-model actuation resulting from the complex motion patterns. Recent studies on novel origami design have proven it could efficiently decrease this energy loss and offer a modeled linear performance (Yi et al., 2018a; Yi et al., 2018b), showing the promising perspective.

The sensory feedback for versatile motions of soft robots is also challenging. Many standard non-deformable sensing technologies are unusable, as the strong coupling of bending and material stretching (which takes place simultaneously most of the time) resulting from the actuators’ soft material characteristics makes the sensory output signals hard to distinguish from each other. A popular remedy to this problem is customizing soft strain sensors for the soft actuator to sense the motion. Examples are resistance strain sensors (Russo et al., 2015; Morrow et al., 2016; Shen et al., 2016; Low et al., 2017; Yang et al., 2017), capacitance strain sensors (Bilodeau et al., 2015; Farrow et al., 2017; Bilodeau et al., 2018; Yuen et al., 2018), and optical waveguide sensors (Sareh et al., 2015; To et al., 2015; Zhao et al., 2016; Molnar et al., 2018; Chen et al., 2019), which hold great potential in this field, although they have suffered from limited reliability and repeatability due to soft materials (either the material itself or connections of soft and rigid components) and complicated fabrication methods. Among these soft sensors, optical waveguide sensors work with a different mechanism that could efficiently avoid soft-rigid connections on the sensing part, therefore largely improving the reliability. However, the strong coupling of the bending and stretching output signal and the complex fabrication process remain challenging.

**FIGURE 1:**
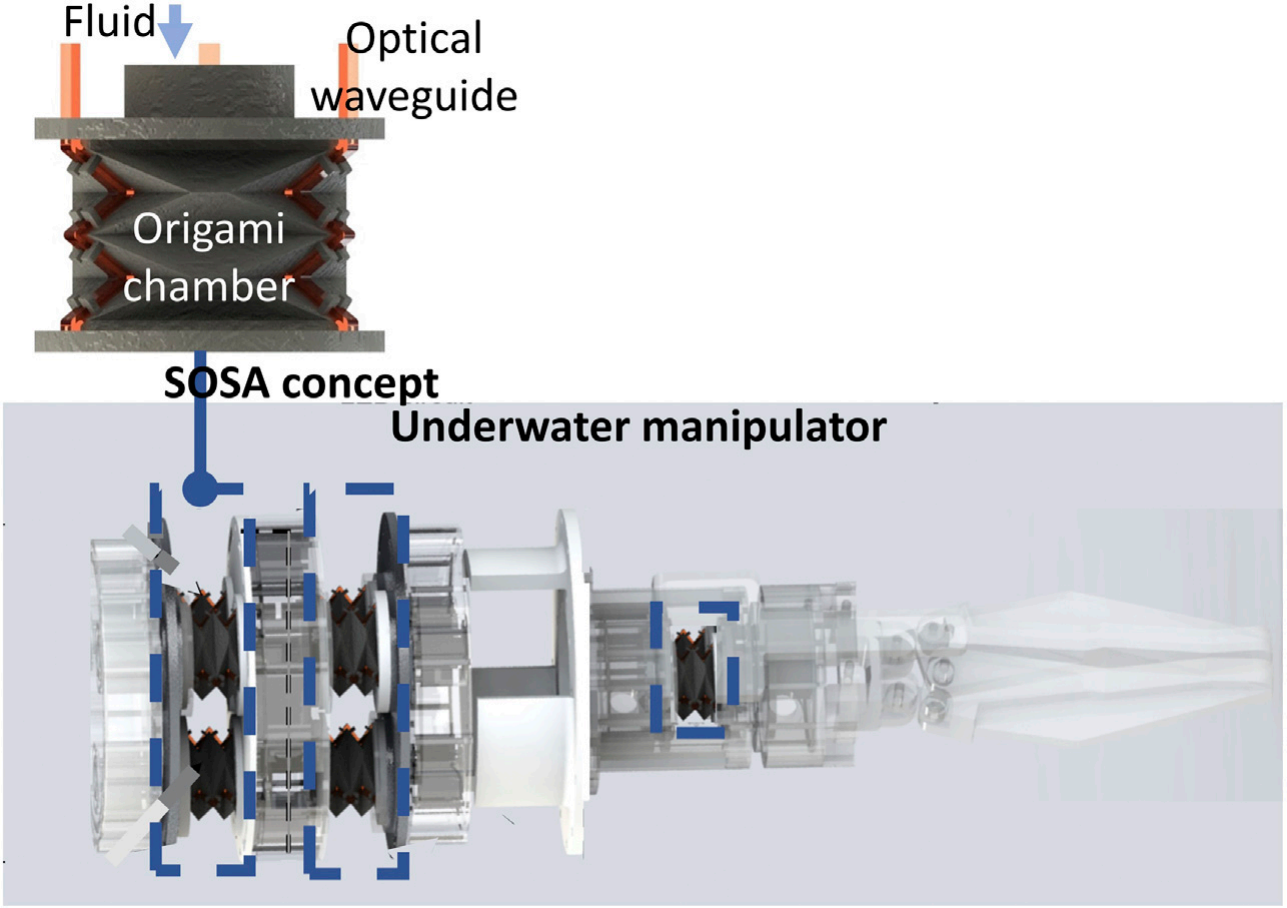
Concept of a Soft Origami Optical-Sensing Actuator (SOSA) with the application for underwater manipulation.

In this paper, we proposed a novel Soft Origami Optical-Sensing Actuator (SOSA) which perfectly integrates the actuating and sensing functions. By newly introducing the origami art, the actuator performance in force output and sensory feedback, and integrative capability, showed significant improvements. The core SOSA design is an origami chamber driven with water and embedded with three specially designed optical soft waveguides, where photodiodes and LEDs are placed on the top and bottom, respectively, for sensory feedback. The proposed actuator could achieve good manipulating and sensing performances in harsh environments with a simplified fabrication process. An underwater manipulator equipped with seven actuators (six for omnidirectional continuum movements, one for grasp) (Figure 1) was developed to showcase the performance of a SOSA actuator.

The main contributions of the paper include: 1) a novel design of an actuating-sensing integrated actuator with large force output and sensing accuracy inspired by origami which enables a superior large payload output and robust and accurate sensing performance by introducing the origami design, significantly facilitating the integration of sensing and actuating technology for wider applications; 2) simplifying the fabrication process for harsh environment applications by investigating the boundary features between optical waveguides and ambient water to remove the external cladding layer of traditional sensors; and 3) proposing a new hybrid underwater manipulator with redundant position feedback for accuracy and dexterous underwater applications.

The paper is organized as follows. *Design Concept, Modeling, and Fabrication Process* section describes the design concept, modeling, and fabrication of the SOSA actuator. *SOSA Actuator Validation Tests* section presents SOSA performance validation tests. Manipulator’s design, motion validation, and sensory feedback are presented in *Hybrid Underwater Manipulator System and Validation Experiments* section.

## Design Concept, Modeling, and Fabrication Process

To address the main contributions on actuator performance and fabrication, two challenges are raised: exploring a sensorized actuator design, which could offer a superior large payload output and robust and accurate sensing performance; and finding an easy and low-cost fabrication method for application in harsh environments, i.e., underwater manipulation.

**FIGURE 2:**
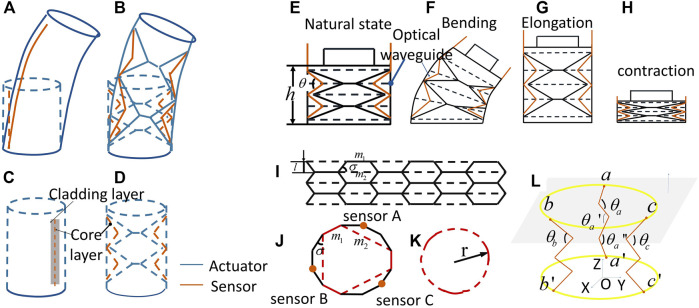
Design concept illustrations of the SOSA actuator: **(A)** traditional soft actuators bending with the soft sensor attached. **(B)** the proposed actuator bending diagram based on origami. **(C)** schematic diagram of structures of the traditional soft actuator. **(D)** schematic diagram of structures of proposed actuator. **(E) (F) (G) (H)** actuator in natural, bending, elongating, and contracting states. **(I)** the deployed actuator structures **(J)** cross-section view of the actuator **(K)** schematic drawing of cross-section inflation **(L)** schematic diagram of SOSA, illustrating the relationship between critical angle and the orientation of the actuator.

The main challenge for the first objective is on the novel design of the actuator and sensor. Traditional soft actuators usually obtain sensory feedback by attaching soft sensors, as shown in Figure 2A. The working mechanism of the actuator utilizes materials’ stretching to generate bending motions, where bending and elongation both result from the nonlinear stretching of the elastic materials. Complicated analysis is involved to model and sense the actuator performance. In addition, the output force and durability of the actuator are also involved. The proposed actuator concept (Figure 2B) uses unique folding structures to release the nonlinear materials’ inflation with negligible stretching (wall thickness = 0.7 mm). This feature may contribute to a large output and accurate sensing performance.

As waterproofing is a critical issue in underwater applications, we chose to use optical waveguide, as only the soft waveguide needs to be exposed to water and all the electronics can be separated and sealed well, which largely reduces system design complexity. As for the actuator fabrication, generally, the optical waveguide needs at least two layers (Figure 2C): one core layer to transmit light and one cladding layer to provide a lower refractive index and protection for the core layer. This well-developed method (Zhao et al., 2016) involves a very complex fabrication process, especially in making the soft optical waveguide. Distinctively, in our proposed SOSA actuator design, only one silicone layer is used for the waveguide (Figure 2D). As the proposed actuator is applied underwater, where the material’s refractive index is larger than water (1.33), water could be naturally used as the cladding layer. In this case, the fabrication process of the optical waveguide can be simplified.

### Design Concept and Modeling

The novel design of the actuator with large force and a precise sensing output is fundamentally facilitated by the new origami-inspired actuating-sensing mode. Two main objectives are required by the origami-inspired actuator design. These involve using origami folding structures to generate robust contraction, extension, and passive bending instead of material inflation which lacks actuation linearity and strength. Also required is the use of origami folding structures to offer a deployable sensing mode instead of material inflation which couples bending and stretching motions. Based on these two objectives, this section investigates the origami-inspired design concept and mechanisms in detail as a general reference.

Origami, the ancient art of paper folding, has uses in geometrical space extension and smart programmable motion mechanisms, which have led to various applications in erospace engineering, architecture, and robotics. Taking inspiration from basic research on origami patterns (Buri and Weinand, 2008; Stavric and Wiltsche, 2014; Liu et al., 2015; Cai et al., 2016; Morgan et al., 2016; Wang et al., 2018), our design was developed based on the Yoshimura pattern (Buri and Weinand, 2008; Morgan et al., 2016). Unlike rigid origami (Stavric and Wiltsche, 2014; Cai et al., 2016), by allowing ridges’ distortion and slight facets’ deformation, utilizing its high foldability among units, the proposed design could offer multiple motion patterns and unique sensing modes simultaneously. Figures 2E–H plot the origami chamber in a natural state (no external force exerted on it), bending state (external force unevenly exerted on it), elongation state, and contraction state. In the linear states (no bending), the length of chamber h could be presented as

$$h=4l\sin\left(\frac{\theta }{2}\right),$$ (1)

where θ is the intersection angle of two facets of the origami design, which could also be presented as the intersection angle between ridges of optical waveguides. l is the geometrical length in the origami unit as labeled in Figure 2I. Maximum elongation length $h_{\max}$ and $h_{\min}$ could be achieved,

$$\left\{ \begin{array}{l} h_{\max}=4l\\ h_{\min}=\left(2n+2\right)t \end{array}\right.,$$ (2)

where n is the numbers of origami units vertically and t is the wall thickness. As facts and ridges deform during actuation, which has a large effect on the minimum state, $h_{\min}$ is calculated by adding the wall thickness of layers.

The output force generated at the cross section f is presented as

$$f=P\pi r^{2},$$ (3)

where P is water pressure and r is the radius of the chamber cap (Figure 2K), which mainly depends on the design of origami parameters, illustrated in Figure 2J. The origami chamber could be fully deployed into a plate, as shown in Figure 2I. The cross-section of the actuator cap is a polygon which could be radially distorted under actuation. The perimeter of the actuator cross-section c is

$$c=3\left(m_{1}+m_{2}\right),$$ (4)

therefore, the radius of the cross-section under actuation could be approximated to

$$r=\frac{c}{2\pi }=\frac{3\left(m_{1}+m_{2}\right)}{2\pi }.$$ (5)

All states of the actuator, including contraction, elongation, and bending, could be sensed by three optical waveguides labeled with sensor A, B, and C on the surface of the origami. The unique mechanism of the sensor is introduced; as shown in Figure 2L, when the intersection angle θ becomes smaller, the upper and lower surface of the optical waveguide will have a higher contact area. This area provides a “shortcut” for the light, as the light can propagate straight toward the photodiode. In this case, the smaller the intersection angle θ, the larger the photodiode’s output will be. Relatively large facet deformation would happen on the maximum deploying and minimum folding states, which may have an effect on the relations of θ and photodiode’s output. This effect could be weakened by tuning the boundaries and thicknesses of ridges and walls to decrease the deformation on the facets. Therefore, by reading the signals from three sensors, i.e., $\theta_{a},\theta_{b},\theta_{c}$, we could obtain the pitch and roll angles of the actuator with the following models.

Considering the output force and motion are transmitted by the two caps of the actuator which are mounted on rigid parts, we assume these two caps as rigid plates. Hence, points a',b',c', as well as points a,b,c, are always in one plane,

$$a'\left(-r,0,0\right);b'\left(\frac{r}{2},\frac{-\sqrt{3}r}{2},0\right);c'\left(\frac{r}{2},\frac{\sqrt{3}r}{2},0\right),$$ (6)

$$a\left(x_{a},y_{a},z_{a}\right);b\left(x_{b},y_{b},z_{b}\right);c\left(x_{c},y_{c},z_{c}\right),$$ (7)

where r is the radius of the actuator. The pitch angle α and roll angle β of actuator could be presented as

$$\left\{ \begin{array}{l} \sin\alpha =\frac{\left|\vec{n_{x}}.\vec{a'a}\right|}{\left|\vec{n_{x}}\right|.\left|\vec{a'a}\right|}=\frac{\left|\vec{n_{x}}.\vec{b'b}\right|}{\left|\vec{n_{x}}\right|.\left|\vec{b'b}\right|}=\frac{\left|\vec{n_{x}}.\vec{c'c}\right|}{\left|\vec{n_{x}}\right|.\left|\vec{c'c}\right|}\\ \sin\beta ==\frac{\left|\vec{n_{y}}.\vec{a'a}\right|}{\left|\vec{n_{y}}\right|.\left|\vec{a'a}\right|}=\frac{\left|\vec{n_{y}}.\vec{b'b}\right|}{\left|\vec{n_{y}}\right|.\left|\vec{b'b}\right|}=\frac{\left|\vec{n_{y}}.\vec{c'c}\right|}{\left|\vec{n_{y}}\right|.\left|\vec{c'c}\right|} \end{array}\right.,$$ (8)

where

$$\vec{n_{x}}=\left(0,1,0\right);\vec{n_{y}}=\left(1,0,0\right),$$ (9)

Vectors in three directions are

$$\left\{ \begin{array}{l} \vec{a'a}=\left(x_{a}+r,y_{a},z_{a}\right)\\ \vec{b'b}=\left(x_{b}-\frac{r}{2},y_{b}+\frac{\sqrt{3}r}{2},z_{b}\right),\\ \vec{c'c}=\left(x_{c}-\frac{r}{2},y_{c}-\frac{\sqrt{3}r}{2},z_{c}\right) \end{array}\right.$$ (10)

Based on the consistent features of origami units,

θa=θa'=θa''=θ, (11)

where θa,θa',θa'' are the intersection angles between ridges of optical waveguides. Geometrically, we have

$$\left|\vec{a'a}\right|=4l\sin\frac{\theta_{a}}{2},$$ (12)

Substituting (10) and (12) into (8),

$$\left\{ \begin{array}{l} \sin\alpha =\frac{\left|y_{a}\right|}{4l\sin\frac{\theta_{a}}{2}}=\frac{\left|y_{b}+\frac{\sqrt{3}r}{2}\right|}{4l\sin\frac{\theta_{b}}{2}}=\frac{\left|y_{c}-\frac{\sqrt{3}r}{2}\right|}{4l\sin\frac{\theta_{c}}{2}}\\ \sin\beta =\frac{\left|x_{a}+r\right|}{4l\sin\frac{\theta_{a}}{2}}=\frac{\left|x_{b}-\frac{r}{2}\right|}{4l\sin\frac{\theta_{b}}{2}}=\frac{\left|x_{c}-\frac{r}{2}\right|}{4l\sin\frac{\theta_{c}}{2}} \end{array}\right..$$ (13)

In Equation 13, $\theta_{a},\theta_{b},\theta_{c}$ are the intersection angles between ridges of optical waveguides, which are achieved by sensor signals. l and r are constants decided by the origami design. Therefore, to get the pitch angle α and roll angle β of actuator, we need to calculate values of $x_{a},y_{a}$.

In the top plane where sensor A, B, and C are evenly distributed, we have

$$\left|\vec{ab}\right|=\left|\vec{bc}\right|=\left|\vec{ca}\right|=\sqrt{3}r.$$ (14)

By substituting (10) into (14),

$${\left(y_{b}-y_{a}\right)}^{2}+{\left(x_{b}-x_{a}\right)}^{2}={\left(y_{c}-y_{b}\right)}^{2}+{\left(x_{c}-x_{b}\right)}^{2}={\left(y_{c}-y_{a}\right)}^{2}+{\left(x_{c}-x_{a}\right)}^{2}=3r^{2}.$$ (15)

From Equations 13, 15, we will get the coordinate of $a\left(x_{a},y_{a},z_{a}\right)$ to substitute into (8). Therefore,

$$\left\{ \begin{array}{l} \sin\alpha =\frac{\left|y_{a}\right|}{4l\sin\frac{\theta_{a}}{2}}\\ \sin\beta =\frac{\left|x_{a}+r\right|}{4l\sin\frac{\theta_{a}}{2}} \end{array}\right..$$ (16)

From Equation 16, the bending angle of the actuator in pitch and roll directions could be calculated based on the output signals from sensor A, B, and C. The length of the actuator under contraction and elongation could be obtained with the same three signals. It is noted that slight deviation between the model and real circumstance may be generated in the maximum deploying and minimum folding states, as geometrical parameters of origami may vary due to the facets’ deformation. This deviation could be ignored as the working range of the actuator generally does not include these two states.

### Fabrication Process

**FIGURE 3:**
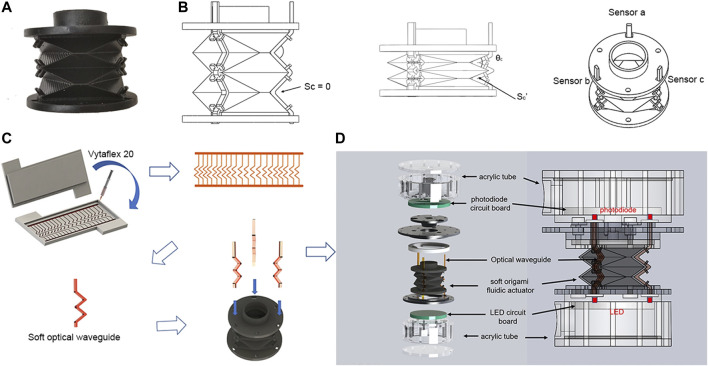
Fabrication process of the SOSA actuator. **(A)** prototype of the soft origami optical sensing actuator. **(B)** schematic drawing of SOSA. $S_{c}$ is the contact area between the upper and lower surfaces of the optical waveguide. **(C)** soft optical waveguide fabrication process. **(D)** SOSA exploded view and assembly.

Fabrication of the SOSA actuator is considerably simplified with the following four steps:

1) The soft origami actuator shown in Figure 3A is fabricated by injection molding with the following parameters: wall thickness t=0.7 mm, number of origami pattern n=2, and origami structure parameters $m_{1}=3.37mm,m_{2}=22.37\;mm,\sigma =35.55^{\circ },h_{natural}=13\;mm$. Polyurethane rubber materials (Hei-Cast 8400, Hardness 60 A) are chosen for their flexible but inflatable features to ensure the folding and deployment of the actuator.
2) Each actuator has three optical waveguide paths and a corresponding housing mechanism for placing the soft waveguide. (Figure 3B) The bottom side of the actuator is permanently sealed, while the top side has a sealing sleeve as inlet and outlet.
3) The fabrication process of the soft waveguide is shown in Figure 3C. A transparent urethane rubber (VytaFlex 20, Smooth-On Inc.) (refractive index equals to 1.461) has been chosen as the soft waveguide material. The molding process is first 3D printed. The liquid urethane rubber is injected into the 3D printed molds. After the silicone is cured, the soft waveguide is removed and cut into the desired shape.
4) Three soft optical waveguides are placed at the surface of the soft origami actuator. Finally, all components are assembled and sealed. As shown in Figure 3D, photodiodes and LEDs are sealed separately in two acrylic tubes which are separately mounted on the top and bottom of the actuator. Note that the photodiodes and LEDs are placed perpendicularly to the acrylic and the viewing angle of LEDs is $18^{\circ }$ to reduce propagation loss.

## SOSA Actuator Validation Tests

In this section, a preliminary test on validating the actuating-sensing concept of the SOSA was conducted. Then a series of overall actuator performance, including the output force, motion range, sensing accuracy, performance repeatability, and reliability of the SOSA actuator were tested to validate the models mentioned in *Design Concept and Modeling* section.

**FIGURE 4:**
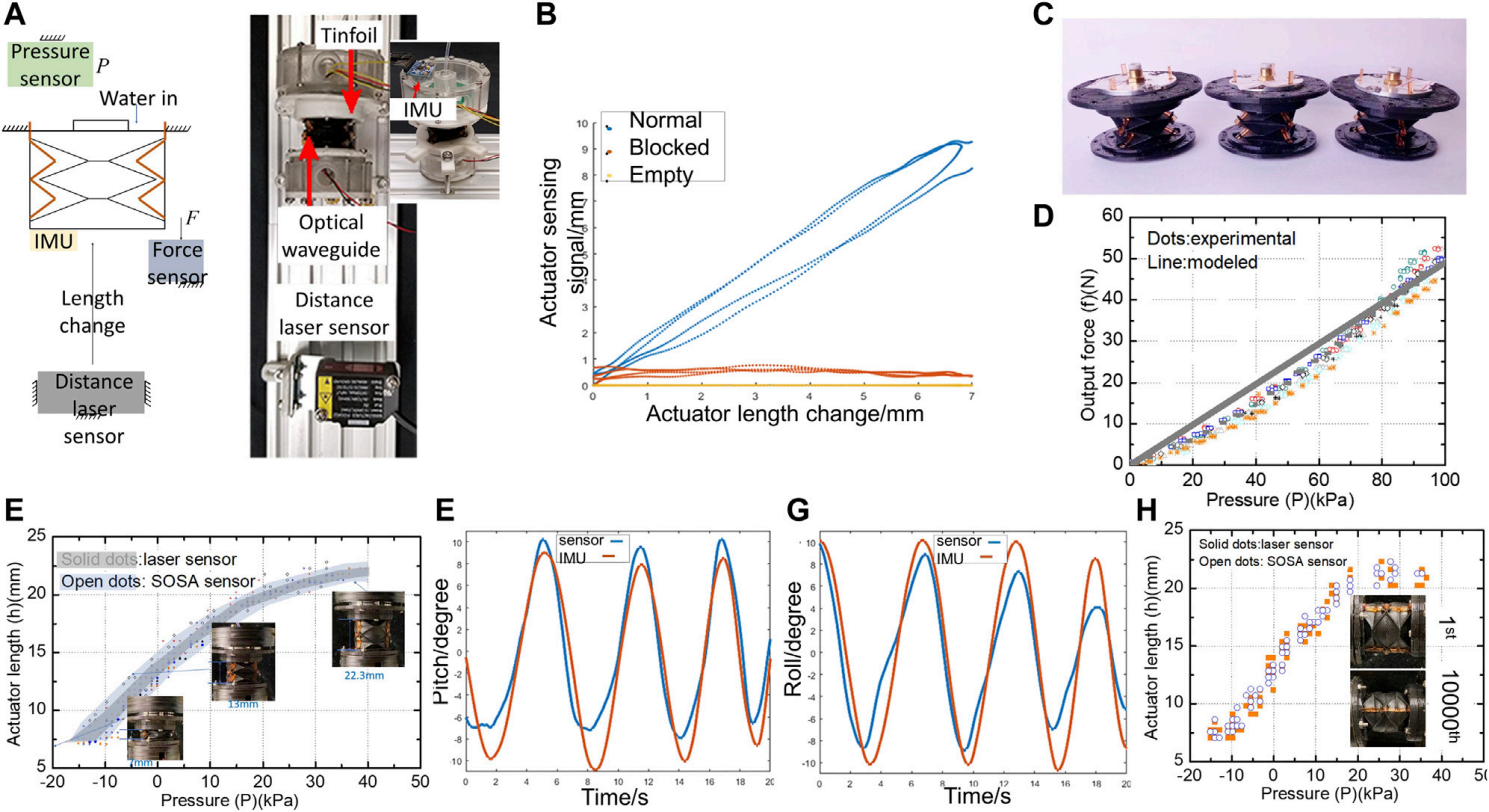
**(A)** schematic diagram and photo of the experimental platform. **(B)** SOSA concept validation test results. **(C)** Three SOSA actuators for repeatability test. **(D)** SOSA isometric test and repeatability test results. **(E)** SOSA actuating-sensing test in free space with sensor signals plotted. **(F) (G)** SOSA bending tests of pitch and roll. **(H)** actuator states comparison before the cycling test and after 10,000th cycles.

The experimental platform, together with its schematic diagram, was plotted in Figure 4A for the above-mentioned tests. In this platform, one cap of the SOSA actuator was fixed to the platform, while the other cap could be set in free space or fixed to the platform connecting to the force sensor. Pressure sensor, force sensor, distance laser sensor (HG-C1100, Panasonic), and 6-DOF Inertial measurement unit (IMU) were used for returning values of water pressure P, actuator output force F, actuator length change, and bending pitch α/roll β angles, respectively.

### Actuating-Sensing Mechanism Validation

A preliminary validation test for the feasibility of the actuating-sensing integrated mechanism was carried out. Based on the actuating and sensing concepts in *Design Concept, Modeling, and Fabrication Process* section, three groups of experimental conditions were set to do the comparison and validate the sensing mechanism under linear actuation: 1) Normal: one optical waveguide was placed as the proposed design; 2) Blocked: one waveguide has tinfoil placed between the upper and lower surface of the waveguide so that there is no “shortcut” when these two surfaces get closer; 3) Empty: the last waveguide path is empty.

During the test, the SOSA actuator was mounted at the photodiode side while the LED side could move axially. The actuator was actuated with water in and out to elongate and contract. The change lengths were measured by a laser distance sensor (HG-C1100, Panasonic) which was placed at the LED side.

The testing results are shown in Figure 4B, which indicate that the actuator could contract and elongate under actuation. Furthermore, according to the feedback of sensor signals, compared to the Empty (photodiode output is 0) and Blocked (photodiode output is constant) ones, only the Normal one returns efficient signals indicating the length change of actuators. Therefore, significant conclusions could be drawn that the actuating-sensing mechanism has been well justified.

### SOSA Isometric Test and Repeatability Test

The correlations of output force f and water pressure P were investigated in the isometric test. In the isometric state, the actuator length was a constant. Two caps were fixed to the experimental platform. We recorded the output force generated at the cap by increasing the inlet water pressure from 0 Kpa to 100 Kpa. Additionally, following the isometric tests’ procedure, three actuators (Figure 4C) with the same dimensions were tested, each repeated thrice, to validate the performance repeatability of actuator.

Figure 4D shows the results on the actuators’ natural length h=13 mm. According to the experimental results, the performance repeatability of the SOSA actuator could be validated from two aspects. One is that good repeatability is shown in three repeated tests of each actuator. The other one is that good repeatability is shown in three actuators tests. In addition, a relatively good linearity between the output force and water pressure is presented, showing agreements with the model (3). Maximum force was achieved on higher water pressure P=100 Kpa compared to the traditional piston (*Festo Pneumatic Cylinde*r (DSNU-16-25-P-A and Festo, 2020), force/weight = 50 N/0.1 Kg); the force to weight ratio of our proposed actuator is much larger reaching to 50N/0.01Kg.

### SOSA Actuating-Sensing Test in Free Space

In the free space state, one cap of the actuator was fixed to the experimental platform, while the other cap was set in free space. The motion range of the actuator was tested by recording the relations between water pressure P and actuator length h. During the test, the length h and bending pitch α/roll β angles were separately returned by the distance laser sensor and IMU for benchmarking to validate the accuracy of SOSA sensor under actuation.

The relations of length and pressure are shown in Figure 4E. Compared to the commercial soft actuator with nonlinear elastic materials which are hard to actuate under 50 Kpa (Yi et al., 2018), the SOSA actuator could easily complete the whole motion range using no more than 40 Kpa. Figure 4E plots the minimum, natural, and maximum length of the SOSA actuator, which indicates that it has a full travel distance of 15.3 mm, more than 100% its natural length. In this test, the sensor signals in detecting length were presented with a blue area. The test results show that the blue area (SOSA sensor) highly overlaps with the gray area (benchmark laser sensor). Significant conclusions could be drawn that the proposed SOSA actuator could obtain a large actuating force output in very low working pressure and high sensing accuracy. This excellent actuating-sensing performance means the SOSA actuator can be applied to more integrative and in harsh environment applications.

SOSA bend sensing tests were conducted in the experimental platform shown in Figure 4A. Different from the above tests, in this setup, a 6-DOF Inertial measurement unit (IMU) was mounted on the top cap of a photodiode tube to read the pitch and roll angles of the actuator with time varying for calibrating the SOSA sensor A, B, and C sensing accuracies. In this setup, one cap of the SOSA actuator was fixed on the platform, while the other cap was manually rotated to generate real-time varying bending motion. Test data could be captured by actuating the actuator, resulting in bending motions. The signals recorded by the optical-sensing actuator and IMU are shown in Figure 4F and Figure 4G, with the RMS error of pitch $2.2^{\circ }$ and the RMS error of roll $2.63^{\circ }$. The results indicate that higher accuracy could be achieved compared to our previous work (Shen et al., 2016), showing relatively good following feedback which could totally satisfy the current application. Slight deviation was generated at the maximum deploying and minimum folding states due to the unmodeled facets deformation. This deviation will be decreased by improving the origami design and signal processing in the future.

### SOSA Performance Reliability Test

The performance reliability of the actuator was validated with a cycling test. In this test, the actuator was required to complete 10,000 cycling movements in the full motion range with a frequency of 0.3 Hz. One cap of the actuator was fixed at the experimental platform, with the other one in free space. Firstly, water was pumped into the actuator, resulting in maximum elongation. Secondly, the water was drawn to maximumly contract the actuator. The cycling test was performed by repeating this process with a frequency of 0.3 Hz. Figure 4H shows the performance comparison of the actuator before test and after test, which indicates that the performance of the actuator was still maintained over 10,000 cycles. Therefore, the performance reliability of the actuator was validated. Further iterations on life span and endurance will be continued in future works.

## Hybrid Underwater Manipulator System and Validation Experiments

### Manipulator Design and System Setup

**FIGURE 5:**
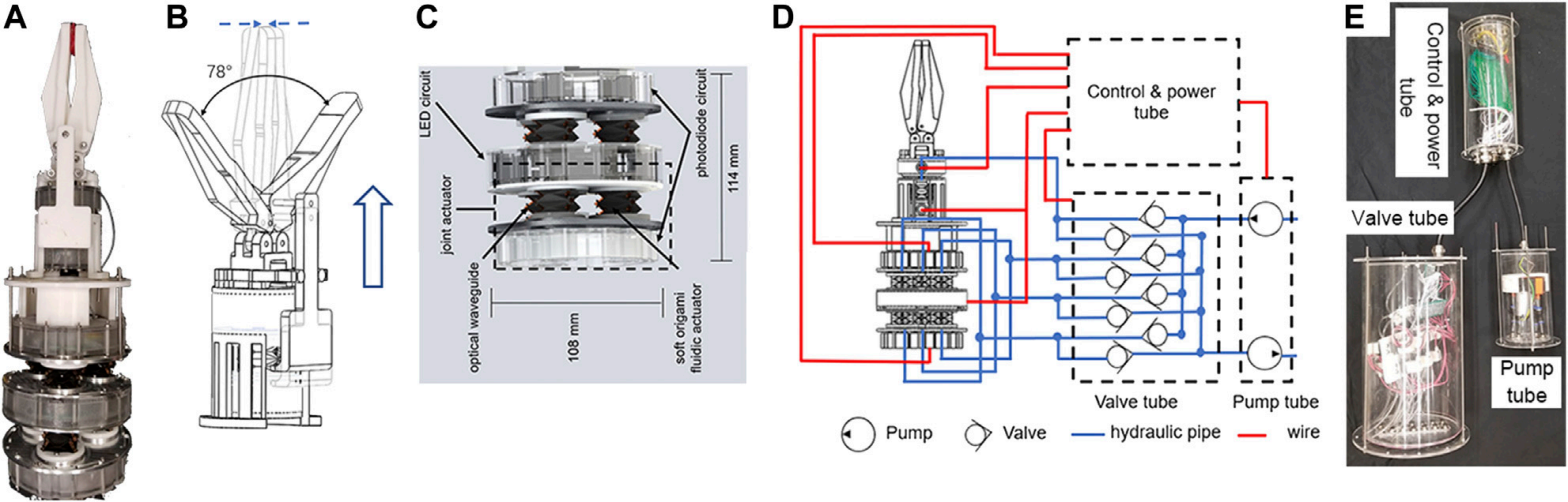
**(A)** The proposed hybrid underwater manipulator. **(B)** Schematic drawing of the gripper. Elongation of the actuator will close the gripper. **(C)** Hybrid underwater manipulator joint assembly. **(D)** Schematic drawing of the hydraulic control system. **(E)** The proposed hydraulic control system.

The SOSA actuator design was applied for the underwater manipulator to showcase its performance. By using the SOSA actuators (Figure 5C) in complex underwater environments, the proposed actuators have the advantage of inherent compliance compared with conventional rigid-bodied robots. Better accuracy and a larger payload can also be achieved. The hybrid underwater manipulator, shown in Figure 5A, consists of two parts: one gripper and one joint. The gripper (Figure 5B) is actuated by one SOSA, which completes the opening and closing of gripper fingers by actuating axially. The maximum opening angle is $78^{\circ }$. Regarding the joint, its diameter and height are 108 mm and 114 mm, respectively. The joint has two sections, each section with three SOSAs mounted triangularly. In addition, two photodiode circuits are sealed inside the middle acrylic tubes. And two LED circuits are sealed in two tubes mounted on the top and bottom of the joint. These components, together with the hydraulic control system, are presented in Figure 5D and Figure 5E, which consists of eight valves, two pumps, and one control unit. For the sake of waterproofing and compactness, all the electronic components are put into acrylic tubes. Six actuators on the joint correspond to six valves, and each valve is connected with two coupled actuators. The remaining two valves are for the actuator on the gripper.

### Hybrid Underwater Manipulator Validation

**FIGURE 6:**
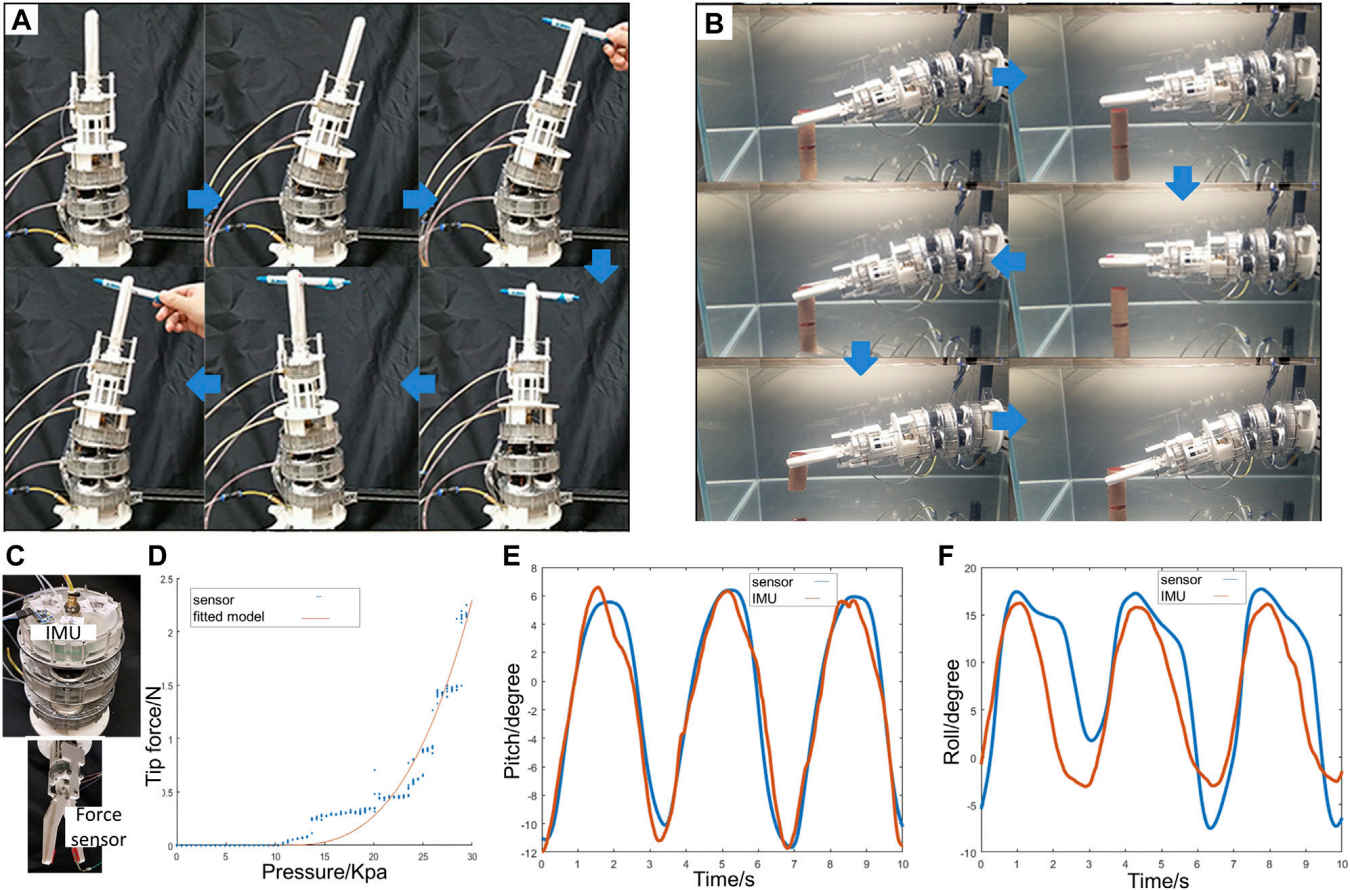
**(A)** Onshore manipulator test. **(B)** Pick and place test underwater. **(C)** Experimental setup for grasping force test and manipulator joint test. **(D)** Grasping force test results. **(E)** Joint test results for the pitch. **(F)** Joint test results for the roll.

A series of tests were carried out to demonstrate the performance of the hybrid underwater manipulator. For the experimental setup, the hybrid underwater manipulator was mounted on a fixed platform and performed different tasks both onshore and underwater. In the on-shore test (Figure 6A), the manipulator was mounted vertically and controlled to grab a pen from an operator, rotate counterclockwise, and return the pen to the operator. In the underwater test (Figure 6B), the manipulator was controlled to perform one pick-and-place task of a cylindrical object. All these movements show high flexibility and high precision positioning, benefiting from the SOSA groups.

### Gripping Force and Joint Test

The gripper’s grasping force was tested for validating the large force output of the SOSA actuator. Figure 6A illustrates the experimental setup, where a force sensor (FSR400, Interlink Electronics) was mounted at the tip of the gripper. As shown in Figure 6D, the grasping force could reach 2.3 N when pressure was only 30 kPa. This force is large in this low actuation pressure, as force generated in this pressure is very limited for the conventional soft actuator (Polygerinos et al., 2015) and compliant gripper (Wang et al., 2013).

As illustrated in the last section, giving three critical angles, the orientation of one SOSA can be calculated. In the manipulator’s joint, the normal vector of each section is in parallel with the corresponding SOSA. In other words, one section’s orientation can be obtained by only one of the three SOSAs. This redundant sensing feedback mode could largely increase system robustness and accuracy. In order to validate the position feedback, an IMU was mounted on the top photodiode tube for benchmarking (Figure 6C). The test results were shown in Figure 6E and Figure 6F, exhibiting the RMS error of pitch in $1.34^{\circ }$ and the RMS error of roll in $5.1^{\circ }$. The results show the same tendency with actuator bend sensing tests. As a short conclusion, this actuating-sensing mode with redundant sensing feedback could largely improve the robustness and accuracy of manipulations, which are offered by the SOSA actuators.

## Conclusion and Future Work

A novel soft origami optical-sensing actuator was proposed in this paper, with concept design, fabrication, modeling, and application presented in detail. Taking advantage of the novel structure of the soft origami actuator, the new actuating-sensing mode on elongation, contraction, and bending motions was implemented in actuator and sensor integration. This new mode has been proven to exhibit superior performance both on an actuation level and sensor level. For the actuator, the larger output could be achieved by introducing the origami design. While for the sensor, more precise feedback signals were provided by the optical sensing method. Additionally, the contribution on fabrication process was also made to simplify the sensor layers. With all these merits, a hybrid underwater manipulator with a novel design on highly redundant actuation and sensing were manufactured with seven SOSA actuators. A series of tests and demonstrations were performed onshore and underwater, showing robust and accurate performance.

In the future, further work on origami design and analytical models will be made to improve the performance and accuracy of SOSA. The position feedback will also be used to form a close-loop control for the hybrid underwater manipulator. Modular design will also be carried out for the manipulator’s joint.

## Data Availability

The original contributions presented in the study are included in the article/Supplementary Material, further inquiries can be directed to the corresponding authors.
